# Harnessing human genetics and stem cells for precision cardiovascular medicine

**DOI:** 10.1016/j.xgen.2023.100445

**Published:** 2023-11-27

**Authors:** Arianne Caudal, Michael P. Snyder, Joseph C. Wu

**Affiliations:** 1 Stanford Cardiovascular Institute, Stanford University School of Medicine, Stanford, CA 94305, USA; 2 Division of Cardiovascular Medicine, Department of Medicine, Stanford University School of Medicine, Stanford, CA 94305, USA; 3 Department of Genetics, Stanford University School of Medicine, Stanford, CA 94305, USA; 4 Greenstone Biosciences, Palo Alto, CA 94304, USA

**Keywords:** genomics, cardiovascular disease, iPSC, stem cell cardiomyocytes, heart

## Abstract

Human induced pluripotent stem cell (iPSC) platforms are valuable for biomedical and pharmaceutical research by providing tissue-specific human cells that retain patients' genetic integrity and display disease phenotypes in a dish. Looking forward, combining iPSC phenotyping platforms with genomic and screening technologies will continue to pave new directions for precision medicine, including genetic prediction, visualization, and treatment of heart disease. This review summarizes the recent use of iPSC technology to unpack the influence of genetic variants in cardiovascular pathology. We focus on various state-of-the-art genomic tools for cardiovascular therapies—including the expansion of genetic toolkits for molecular interrogation, *in vitro* population studies, and function-based drug screening—and their current applications in patient- and genome-edited iPSC platforms that are heralding new avenues for cardiovascular research.

## Graphical abstract

**Figure:**
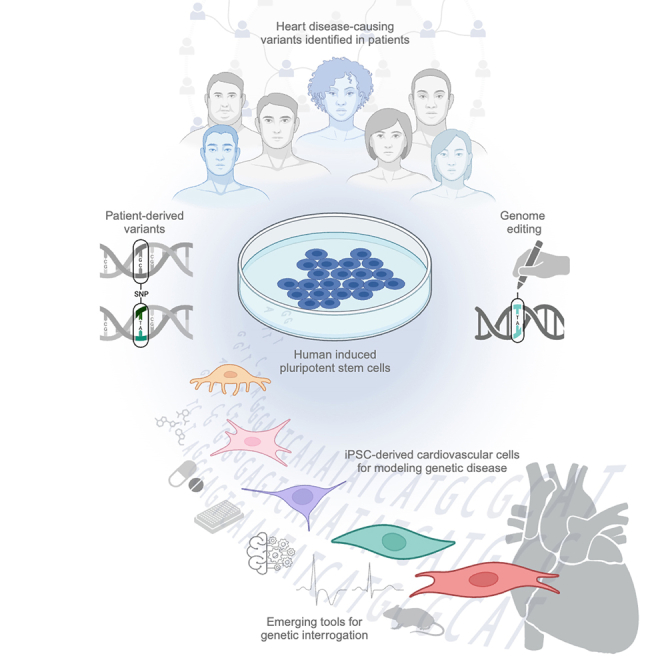

## Teaser

Caudal et al. review emerging molecular technologies used to unpack the influence of genetic variants in the heart. The authors focus on applications of genetic toolkits in human induced pluripotent stem cell models for the prediction, visualization, and treatment of cardiovascular diseases.

## Introduction

**Figure 1:**
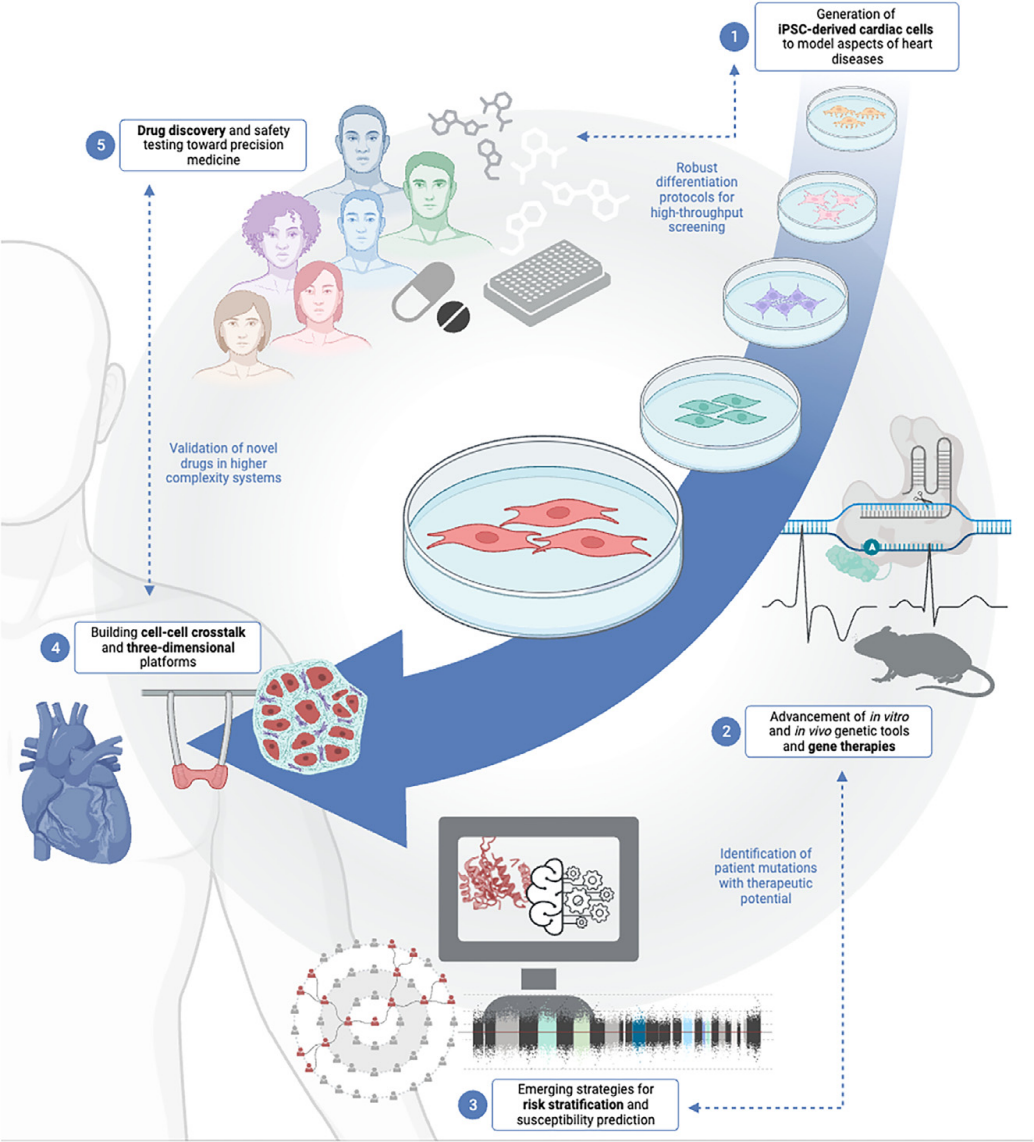
Emerging technologies to propel precision medicine of genetic cardiovascular diseases forward Recent advancements in iPSC technology enable new insight into genetic cardiovascular diseases. In this review, we highlight novel innovations in (1) the generation of iPSC-derived cardiac cells for disease modeling, (2) the expansion of genetic tools and therapies, (3) the prediction of disease-linked genetic variants and patient response risk using functional and computation methods, (4) 3D and multi-cellular platforms, and (5) function-based pharmacogenetic testing and discovery. We provide perspective into how these complementary advancements (blue text) will continue to drive the future of cardiovascular precision medicine. Created with Biorender.com.

Cardiovascular diseases—congenital and acquired disorders of the vasculature, myocardium, and conduction system—are a leading cause of death worldwide.^1^ Largely determined by heritability and epigenetic factors, our understanding of cardiovascular disease risk and causality has expanded alongside technological advancement in genomic and transcriptomic tools. The rise of population studies using genome-wide association studies (GWAS), whole-genome sequencing, and whole-exome sequencing has been instrumental in identifying potential disease-related loci and single-nucleotide polymorphisms (SNPs).^2^ Although it provides valuable insight into the genetic architecture of complex diseases, as a standalone approach genetic screening has limited clinical utility for patient prognosis^3^ as the identified risk variants may fail to fully explain genetic heritability for human disease, individually or collectively.^4^ The need for robust and patient-relevant disease models in clinical genetics has been met by innovations in induced pluripotent stem cell (iPSC) technology, which provides a functional platform for the delineation of genotype-phenotype associations and an unprecedented opportunity to understand population-relevant genetic diversity (Figure 1).

**Figure 2:**
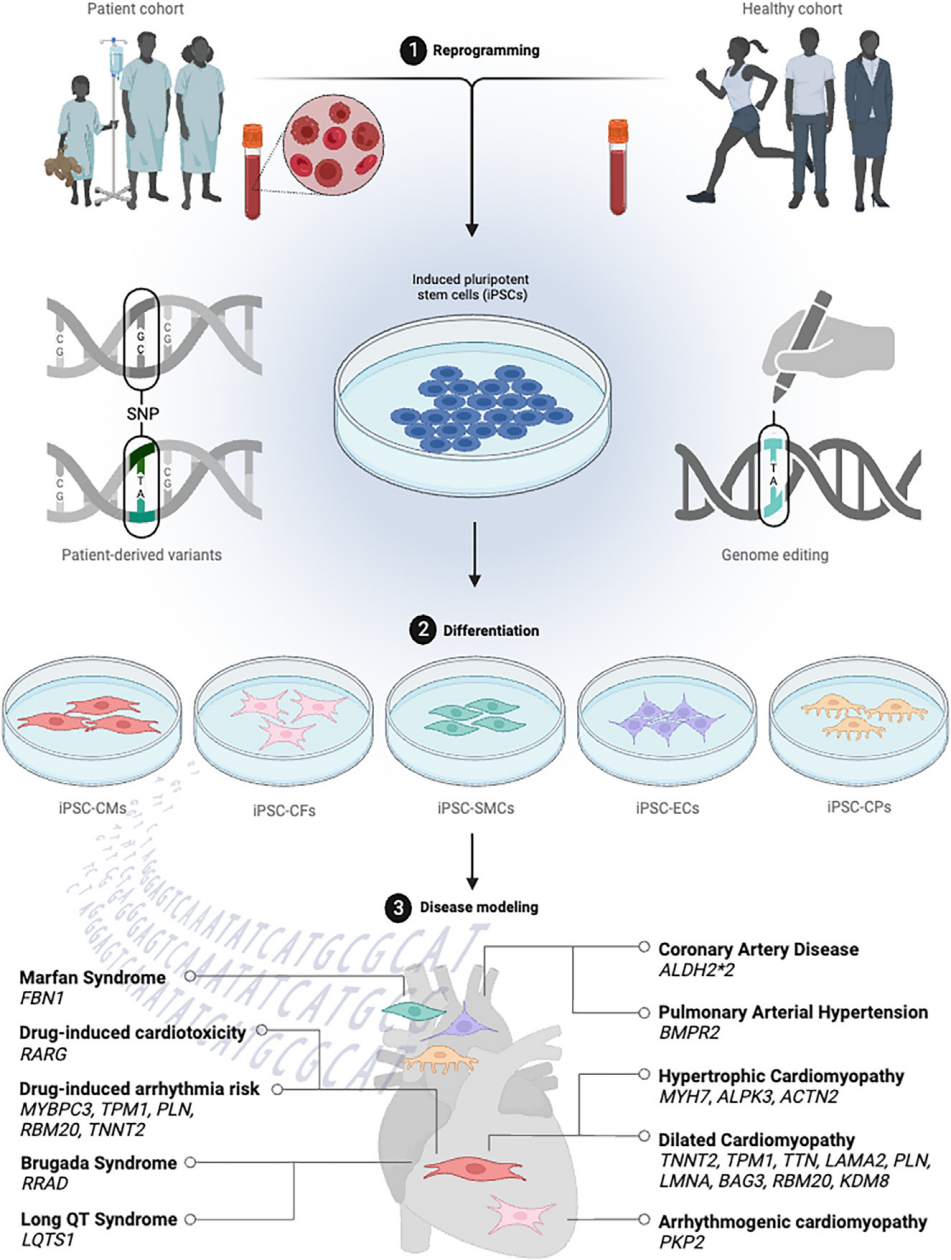
Modeling of cardiovascular genetic diseases using human iPSC platforms (1) Disease variants can be modeled *in vitro* by reprogramming of iPSCs from the somatic cells of patients or healthy controls. Generation of iPSCs maintains the genetic integrity of disease, such as single-nucleotide variants, directly from patients; otherwise, they can be induced using genome editing on healthy control iPSCs. (2) Protocols for the differentiation of iPSCs into specialized cell types such as iPSC-CMs, iPSC-derived cardiac fibroblasts, iPSC-derived smooth muscle cells, iPSC-derived endothelial cells, and iPSC-derived cardiac pericytes are used to assess phenotypes in a dish, (3) providing disease modeling of diverse genetic cardiovascular diseases. Created with Biorender.com.

The heart is an anatomically complex organ that requires exquisite orchestration of heterogeneous cell populations to enable continuous contraction and relaxation across different physiological conditions.^5^ In cardiomyocytes (CMs), the sarcomere is comprised of calcium-dependent regulatory thin filaments, myosin-based force-generating thick filaments, and Z-discs.^6^ Contraction of the sarcomere is coordinated by the propagation of electrical depolarization of the cell membrane by ion channels and calcium-handling machinery.^7^ The nonmyocytes in the heart regulate a multitude of cellular processes, including vascular tone, mechanosensing, and angiogenesis. The direct and indirect communication between specialized cell populations govern molecular responses to physiological and pathological stress.^8^^,^^9^ Human iPSCs can be expanded indefinitely and differentiated into a variety of cardiovascular cell types—including CMs,^10^ endothelial cells (ECs),^11^ cardiac fibroblasts (CFs),^12^ smooth muscle cells (SMCs),^13^ and cardiac pericytes (CPs)^14^—to dissect the molecular underpinnings of heart failure (Figure 2). Given their capacity to recapitulate disease- and mutation-level phenotypes, the application of iPSC-derived cells in genomics enables direct differentiation of pathogenic mutations from background genetic noise, identification of novel variants, genotype- and phenotype-guided risk stratification, and patient-specific drug response.^15^

Here, we summarize recent literature emerging in the past 5 years using iPSC platforms in functional genomics for risk prediction, visualization, and treatment of the heart, with emphasis on how these technological advancements have led to new molecular insight in various cardiovascular disease models.

### Prediction of disease-linked genetic variants and patient response risk

A significant challenge in contemporary genetic medicine is the disconnect between the ease of genetic testing and the availability of experimental platforms that can provide meaningful interpretations of the growing list of novel and rare variants. Thus, predictive technologies that can resolve nuanced phenotypes and iPSC validation have co-evolved as a breakthrough combinatorial tool for patient risk assessments. Modern approaches to studying cardiac arrhythmias exemplify these technical developments.

The cardiac action potential is an electrical signal that dictates contraction, pivotal to heart rhythm and blood circulation. At the tissue level, action potentials are clinically assessed by electrocardiogram traces, which monitor the rhythmic depolarization of the atria (P wave) and depolarization (QRS complex) and repolarization (T wave) of the ventricles. A vexing problem in cardiovascular disease is that ventricular arrhythmia can occur in a structurally normal heart.^16^ Such is the case in Brugada syndrome (BrS), defined by an abnormal electrocardiogram signature of ST elevation with successive negative T wave, putting patients at risk for sudden cardiac death.^17^ At the cellular level, an action potential is initiated by the rapid opening of voltage-gated sodium channels. The plateau period is maintained in a depolarized state by the opening of L-type calcium channels (LTCCs) and inward movement of calcium, balanced by the movement of potassium ions outward. The outward movement of potassium ions repolarizes the membrane to the resting voltage, ending the action potential.^7^ Variants in the sodium channel alpha subunit Na_v_1.5 (*SCN5A*) account for nearly 20%–25% of BrS molecular diagnoses, with roughly <5% of cases resulting from variants involved in Na_v_1.5 regulation or encoding calcium or potassium channel subunits. Susceptibility genes for BrS have conventionally been identified using functional candidate approaches. More recently, Belbachir et al. utilized an unbiased approach to investigate a large pedigree with genetically unexplained BrS. Employment of whole-exome sequencing and identity-by-descent analysis led to the discovery of a rare gain-of-function p.R211H variant in the Ras associated with diabetes (RAD) GTPase.^18^ In addition, iPSC-derived CMs (iPSC-CMs) of affected *RRAD* p.R211H family members and knockin isogenic lines had slower spontaneous beat rhythms with slower but longer-lasting electrical conduction, displayed by lower action potential upstroke velocity and longer action potential duration than intra-familial controls and genome-edited controls. Beyond conduction defects, *RRAD* p.R211H displayed structural abnormalities of the CM cytoskeleton hypothesized to negatively impact cell-cell contact and potentially trigger electrical anomalies in BrS.^18^

Manual electrophysiological measurements—such as the traditional patch-clamp method used to understand how electrical currents flow in and out of an individual cell—require a high degree of expertise from a skilled experimenter, resulting in relatively low throughput. To offset this hindrance, data-driven *in silico* predictors and structural modeling of variant location within protein conformations can be used to assess diagnostic hypotheses (Figure 1). These strategies have been elegantly applied to predicting syndromes of abnormal heart rhythm, such as BrS and long QT syndrome, penetrance probabilities using population metrics,^19^^,^^20^^,^^21^ and iPSC-CM experimentally derived machine-learning approaches.^22^ Recent advancements in patient arrhythmic risk stratification include a wider range of genetic perturbations and pharmacological interventions. In this direction, a convolutional neural network (CNN) was trained using action potential waveforms from iPSC-CMs treated with proarrhythmic drugs from healthy donors and genetic knockin of variants leading to arrhythmogenic cardiomyopathies, diseases of the myocardium that affect the heart’s ability to pump. These included hypertrophic cardiomyopathy (HCM) variants (myosin-binding protein C, *MYBPC3* p.R943X and tropomyosin 1, *TPM1* p.K37E) and dilated cardiomyopathy (DCM) variants (RNA-binding motif protein 20, *RBM20* p.R634Q, and phospholamban, *PLN* p.R14del)^23^ in genes affecting sarcomere contractile machinery and calcium homeostasis. The CNN class probabilities mapped each trace to a spectrum from non-arrhythmic to arrhythmic/asystolic *in vitro* phenotypes, and the resultant safety margins more accurately risk-stratified proarrhythmic drugs than previous human-defined adjudication.^23^ Aiding a significant clinical challenge, the CNN platform was also able to quantify the increased risk of drug-induced arrhythmia in cardiomyopathic gene variants. Integrating predictive technologies with large population records, longitudinal follow-up,^24^ and *in vitro* phenotyping data will be critical for future drug development, safety, and efficacy.

### Expansion of genomic tools for cardiovascular modeling and therapy

#### CRISPR-Cas9-mediated gene therapy validated using iPSC-CMs

Genome-editing strategies have revolutionized biomedical investigation, first as a tool to generate research models and second as a therapeutic avenue to treat or prevent disease.^25^ The ability of bacteria and archaea to cleave foreign DNA as part of their adaptive immunity inspired a two-component system, amended for programmable gene manipulation in eukaryotic cells.^26^ This RNA-guided CRISPR-Cas (clustered regularly interspaced short palindromic repeats/clustered regularly interspaced short palindromic repeat-associated proteins) technology has made applications of gene editing more accessible and multifaceted than traditional approaches.^27^^,^^28^ In the conventional CRISPR-associated protein 9 (Cas9) system, the guide RNA dictates target recognition by Watson-Crick RNA-DNA base pairing. The protospacer adjacent motif (PAM) sequence is recognized by the CRISPR-Cas9 endonuclease, which generates a double-stranded break (DSB) in the DNA that is repaired by non-homologous end joining or by homology-directed repair (HDR) in the presence of a donor DNA template.^26^

The recent development of CRISPR-Cas9 base editors for targeted point mutations can convert a single DNA base pair without needing DSBs and donor DNA templates,^28^ thus emerging as an attractive approach for the correction of genetic diseases, especially monogenic diseases with single-nucleotide variants. Instead, current base editors have a catalytically deactivated Cas9 enzyme fused to DNA deaminases. Adenine base editors (ABEs) catalyze an A>G transition in the PAM strand (T>C change in the target strand), and have notably been applied to therapeutic gene correction of a monogenic myosin heavy chain (*MYH7*) p.R403Q variant in iPSC-CMs^29^ to improve contractility and in murine hearts to prevent HCM onset (Figure 1).^29^^,^^30^ These studies provide the first demonstration of highly efficient single-nucleotide correction in postnatal mammalian CMs.^29^^,^^30^ It remains to be determined whether ABE editing has therapeutic potential to prevent HCM onset for the lifespan of a mouse, in larger mammalian models, and within the context of pre-existing HCM. Still, preclinical strategies such as ABE hold tremendous translational potential for a population of nearly ∼25,000 individuals who have this precise *MYH7* variant.^29^

Prime editing (PE) technology enables the induction of all types of base pair transitions and transversions, small insertions, and deletions.^31^ PE utilizes a Cas9 nickase fused to an engineered reverse transcriptase domain in combination with PE guide RNAs, which specify the target site and the desired edit.^28^ In iPSC-CM models for DCM, PE was used to correct the pathogenic RNA-binding motif protein 20 (RBM20) p.R636S variant with 40% A-to-C editing efficiency. In parallel, the RBM20 p.R634Q mutation was corrected using an ABE strategy at 92% efficiency, which normalized abnormal gene expression and cardiac function in cells and murine hearts.^32^ Advancements in ABE and PE have expanded the applicability of precise genome editing as a viable, therapeutic avenue for a wide variety of monogenic diseases, such as its recent application to iPSC-CM models of Duchenne muscular dystrophy,^33^^,^^34^ thus presenting a novel frontier for cardiovascular research.

#### Genetic engineering for elucidation of protein interactions

CRISPR-Cas9 genetic engineering enables the generation of endogenous fusion enzymes with relative ease, which can be utilized for protein proximity network analysis from living cells and animals.^35^^,^^36^^,^^37^ Proximity proteomics captures a wide breadth of transient, dynamic, organelle-specific processes as they exist in the native cellular environment.^38^^,^^39^^,^^40^^,^^41^ Its recent application to iPSC-CMs and murine hearts has made it possible to describe physiological and pathological processes vital to the cardiovascular system,^42^ including the following: the elucidation of the protein networks spanning between the sarcoplasmic reticulum and transverse tubules,^43^ transcription factor interactions in cardiogenesis,^44^^,^^45^ and isoform-specific caveolin interactions in CMs.^46^

α-Actinin 2 (*ACTN2*, referred to as actinin) is a Z-disc anchoring protein that forms primary scaffolding interactions between the contractile apparatus and cytoskeleton.^47^ In addition, actinin forms secondary interactions that propagate signaling, transcription, and protein homeostasis.^48^ To systematically contextualize the cellular roles of actinin, its protein neighbors were determined with proximity-dependent biotinylation (BioID).^48^ Actinin was fused to the promiscuous biotinylating enzyme, BirA, using CRISPR-Cas9 genetic engineering at the endogenous *ACTN2* locus. Protein expression levels of actinin-BirA fusion protein in iPSC-CMs were similar to endogenous levels of actinin in control iPSC-CMs that did not express BirA. Biotin supplementation and subsequent streptavidin immunocapture followed by tandem mass tag liquid chromatography-mass spectrometry of actinin-BirA and control non-BirA iPSC-CMs led to the enrichment of 324 actinin proximity partners. The combination of genetic tools with high-throughput quantitative proteomics enabled the discovery of actinin-IGF2BP2 interaction, which regulates metabolic transcript localization during pathological sarcomere function.^48^

The pathogenic role of promiscuous actinin protein interactions was elegantly described by the novel c.740C>T (p.T247M) *ACTN2* variant, which recapitulates HCM in patient-derived and CRISPR-induced iPSC-CMs.^49^ Furthermore, in three-dimensional (3D) engineered heart tissues (EHTs), Prondzynski et al. describe prolonged action potential duration and increased LTCC current density that were likely propagated by reduced interaction with mutant actinin.^49^ The interaction between LTCCs and actinin leading to modulation of ion channel activity has been described previously.^50^^,^^51^ Here, the availability of patient-derived iPSC-CM cell and tissue platforms allowed for the testing of diltiazem, an LTCC inhibitor, in the context of c.740C>T *ACTN2* before it was administered to HCM-affected family members to ameliorate the electromechanical phenotype.^49^ Alternatively, CRISPR-Cas9 modeling of c.740C>T *ACTN2* in iPSC-CMs exhibited proteomic alterations in several canonical pathways, including mitochondrial function, sirtuin signaling, protein ubiquitination, myofilament organization, and mRNA stabilization.^52^ Thus, system-wide approaches for dissecting the pleiotropic roles of actinin interactions in documented *ACTN2*-associated cardiomyopathies^53^^,^^54^^,^^55^^,^^56^ will be instrumental for future HCM studies.

In summary, advancements in CRISPR-Cas9 technology provide a versatile genetic platform for disease modeling and gene therapy with integrative applications for system-wide proteomic profiling, which has ushered in a new age for molecular probing of the cardiac microenvironment of human genetic disease.^28^

### Utility of iPSC-derived cells for pharmacogenetic testing and discovery

The development and approval of new cardiovascular drugs have stagnated for two decades, despite heart failure being the leading cause of death worldwide. This clinical hurdle is partly due to the common phenomenon of encouraging early-stage results being frequently reversed by disappointing outcomes in late-stage trials.^57^^,^^58^ Furthermore, access to human cardiac samples was previously limited to primary culture or postmortem autopsy, making the clinical testing of cardiovascular drugs difficult. The use of iPSC-based platforms has created innovative solutions that now provide pre-clinical screening and early-phase drug safety testing to resolve long-standing genetic problems (Figure 1).

Human-based models are advantageous for understanding how SNPs affect drug responses, even in genes whose connection to toxicity may be previously unknown. For example, since its introduction in the 1960s, doxorubicin has been an effective and widely prescribed chemotherapeutic agent.^59^ However, dose-dependent doxorubicin-induced cardiotoxicity (DIC) is well documented in cancer patients, and these cardiotoxic side effects can include arrhythmias, congestive heart failure, and heart transplantation.^60^ GWAS identified the loss-of-function rs2229774 (p.S427L) variant in retinoic acid receptor gamma (*RARG*) that is associated with DIC in patients.^61^ To confirm causality, DIC susceptibility was probed in rs2229774 patient-derived and *RARG* KO iPSC-CMs, which led to increased DNA damage and diminished mitochondrial function. Genetic overexpression of *RARG*, or treatment with the RARG agonist, CD1530, was found to improve cell viability and function without diminishing the anti-cancer efficacy of doxorubicin in human cells and mouse models.^62^ In linking a genetic variant to its drug efficacy, Magdy et al. provide a rationale for both pre-chemotherapy pharmacogenetic testing for rs2229774, and increased cardiac monitoring in cancer patients receiving anthracyclines.^62^

In a hypothesis-driven approach to rescuing the DCM-related heterozygous RBM20 p.P633L variant, all-*trans* retinoic acid (ATRA) was found to stimulate the transcriptional upregulation of *RBM20*, which compensated for cellular deficits in splicing, calcium handling, and contractility. Although the precise molecular mechanism of how ATRA pharmacologically stimulates residual *RBM20* transcription remains unknown, it led to the finding that boosting RBM20 protein levels, stability, or activity may have therapeutic potential.^63^ Similarly, a candidate approach was used by Guo et al. to find a compound that mitigates reactive oxidative species and boosts nitric oxide in iPSC-derived ECs with alcohol dehydrogenase 2 (*ALDH2∗2* rs671) variant, leading to drug repurposing of the sodium glucose cotransporter-2 inhibitor, empagliflozin.^64^

Using an unbiased drug screen in a variety of DCM iPSC-CMs, Perea-Gil et al. assessed a small-molecule library containing 160 kinase inhibitors for their ability to rescue iPSC-CM and EHT contractility, revealing the efficacious combination of Gö 6976 and SB 203580.^65^ At an intersection between precision medicine and “classical” drug screening, this study employs patient-specific material to unearth a tailored therapy with potentially broad applicability to a multitude of DCM patients. Finally, high-throughput screening was demonstrated in an activity-based system that screened 1,022 small-molecule inhibitors in a genetically engineered model for Marfan syndrome (MFS), revealing glycogen synthase kinase 3 beta (GSK3β) as a positive hit among other interesting targets.^66^ Because GSK3β is ubiquitously expressed and has over 500 targets, it may be challenging to target in a translational setting. However, this study illuminates molecular insight into potential combinatorial approaches and optimizations for MFS and other genetic aortic diseases.^66^ As a major step toward precision medicine, studies such as these demonstrate the potential of iPSC-based platforms for clinical trial population screening based on drug responsiveness,^67^ and facile applications of both hypothesis-driven and unbiased drug discovery pipelines for rapid clinical translation.

### New molecular insights into cardiovascular pathogenic variants

As patient-derived iPSCs can recapitulate disease-associated cellular phenotypes *in vitro*, these models have gained traction for studying cardiovascular disease and guiding the refinement of therapeutic approaches. Furthermore, genome editing strategies enable the practical introduction of precise allelic variants and point mutations in a controlled iPSC background, providing a secondary source to model disease.^28^ Here, the cutting-edge techniques previously described are employed to determine causal relationships between genotype and phenotype in various cardiovascular diseases (Figure 2).

### *Modeling dilated cardiomyopathies using iPSC-CMs*

Clinically characterized by an enlarged and poorly contractile left ventricle, DCM has an estimated prevalence of 1:250 adults^68^ and is a leading condition for heart transplant.^69^^,^^70^ End-stage tissue remodeling in DCM results in thinning of the ventricle walls, driving mechanical stress in the myocardium, promoting CM cell death, increased fibrosis, and maladaptive metabolic alterations.^71^ DCM has both monogenic and polygenic disease origins with at least 19 causal gene mutations across various cellular processes, including force transmission and cardiac conduction.^69^^,^^70^^,^^72^ Despite significant progress in elucidating the genetic implications of DCM, its broad cellular underpinnings make prevention strategies and disease-modifying agents challenging to resolve. For example, genetic variants in the cytoskeletal, sarcomeric, mitochondrial, desmosomal, nuclear membrane, and RNA-binding protein genes are linked to the development of DCM, thus showing that diverse genetic inputs lead to similar pathophysiology. Innovative treatment strategies for DCM likely require a comprehensive understanding of how genetic etiology manifests in molecular phenotypes, including distinct DCM-associated genes. Thus, the advent of patient-derived and CRISPR gene-editing tools for the faithful recapitulation of DCM *in vitro* has enabled substantial progress in understanding genetic-phenotypic relationships in this disease. For example, iPSC-CMs were generated from five DCM patients harboring pathogenic mutations in titin (*TTN*, c.73817delC, p.P24606LfsX16), phospholamban (*PLN*, c.40_42delAGA, p.R14del), lamin A/C (*LMNA*, c.967_968delCT, p.L323fs), tropomyosin 1 (*TPM1*, c.688G>A, p.D230N), and laminin subunit alpha 2 (*LAMA2*, c.7074C>A, p.Y2358X). Patient-derived iPSC-CMs demonstrated a contractile and mitochondrial respiration deficit that is rescued by combinatorial treatment of small molecule kinase inhibitors (SMKIs). Induction of activating transcription factor 4 (*ATF4*)-mediated rescue of serine biosynthetic pathways by SMKIs was found to ameliorate contractile and respiratory bottlenecks by replenishing intermediary metabolism in a cell-autonomous and genotype-agnostic manner.^65^ These findings represent a novel therapeutic axis linking the roles played by serine, glycine, and one-carbon metabolism in cardiac physiology and pathophysiology.

Inherited forms of DCM with known causal variants are found in approximately 30% of affected individuals, with the genetic drivers for most cases remaining unknown. Hypotheses beyond the scope of force transduction, myocardial energetics, and conduction are being actively explored. Mutations in B cell lymphoma 2-associated athanogene 3 (*BAG3*) cause monogenic forms of DCM due to its critical role in maintaining the protein quality of mechanically damaged contractile proteins, as it serves as the structural scaffold between F-actin to α-actinin.^73^^,^^74^^,^^75^ Although over 250 human *BAG3* variants have been reported as potentially deleterious in public databases, how and if such mutations initiate disease remains phenotypically understudied. McDermott-Roe et al. employed genome-edited iPSC-CMs in the first exploration of the DCM-linked *BAG3* missense variant (c.1430G>A; p.R477H), which causes proteasome inhibition-mediated fiber disarray and dysregulation of the protein quality stress response by uncoupling protein interactions between BAG3 and HSC/HSP70. Furthermore, lentiviral overexpression of heat shock factor 1 (*HSF1*), a transcription factor that regulates stress response genes, reduced the proteostatic deficits induced by the BAG3 p.R477H variant on myofibrillar organization.^73^

Further highlighting the importance of protein quality control, autosomal dominant mutations in *RBM20* account for approximately 3% of DCM cases and can present with earlier-stage onset and greater severity than *LMNA* or *TTN* mutations.^76^ Genetic engineering of heterozygous and homozygous gain-of-function RBM20 p.R636S allelic mutants by transcription activator-like effector nucleases and CRISPR-Cas9 gene editing yielded iPSC-CMs that exhibited electrophysiological and contractile abnormalities reminiscent of DCM observed in patients. Precise targeting of RBM20 patient mutations in both 2D iPSC-CM monolayers and 3D EHTs helped delineate the complex relationships among RNA biogenesis with excitation-contraction coupling, co-localization of mutant RBM20 with cytosolic P-bodies, and alterations in spliceosome-mediated targets.^76^ Similar to DCM-associated RBM20 p.P633L loss-of-function variant,^63^ RBM20 p.R636S possesses poor nuclear localization potential. Promotion of the protein-protein interaction between pathogenic RBM20 and nuclear import via transportin-3 (TNPO3) was recently shown to alleviate protein mislocalization and defective alternative splicing in RBM20 p.P633L and p.R634Q mutant iPSC-CMs.^77^ Whether the affinity for the RBM20-TNPO3 interaction can be increased with potent pharmacological agents, or whether ATRA is efficacious in the gain-of-function heterozygous RBM20 p.R636S variant, remains to be determined.

Understanding the epigenetic role of histone methyltransferases and demethylases as regulators of cardiac homeostasis through key transcription factors provides a new direction for DCM hypotheses. Specifically, the role of lysine demethylase 8 (*KDM8*) in the repression of T-box transcription factor 15 (*TBX15*)-controlled networks in cardiac NAD+ homeostasis and metabolism was recently illuminated.^71^ Missense mutations in *KDM8* are associated with Coffin-Siris syndrome, which presents with congenital cardiac abnormalities in ∼30% of patients.^78^ Interestingly, *TBX15* was upregulated in *KDM8* null and *PLN* null mice, both developing DCM leading to heart failure, but not in *TTN* mutants that developed only hypertrophy. Overexpression of *TBX15* in iPSC-CMs resulted in the repression of the nicotinamide phosphoribosyltransferase (*NAMPT)* promoter and blunted NAD-activated respiration *in vitro*. Transcriptional findings featuring the KDM8-TBX15 axis were further employed to identify a group of DCM-affected human hearts with profound metabolic derangement. Results suggest that KDM8 epigenetically controls TBX15 to prevent maladaptive metabolic remodeling toward DCM.^71^

### *Modeling hypertrophic cardiomyopathies using stem cell platforms*

HCM is characterized by the abnormal thickening of the ventricular myocardium, creating a hyperdynamic state that leads to an energetic deficit due to increased cardiac output.^79^ Autosomal dominant mutations in *MYH7* and *MYBPC3* genes, which encode the sarcomere thick filament proteins, account for nearly 80% of inherited HCM cases^80^ and were recently tackled with genome-editing tactics in conjunction with iPSC-CM modeling. The dominant negative pathogenic mutation, *MYH7* c.1208G>A (p.R403Q), is a well-studied heterozygous missense variant that causes early-onset HCM and progressive myocardial deterioration. This variant weakens the critical protein-protein interaction between the myosin head with MYBPC3, ultimately leading to augmented sarcomere contractility. Using ABEs, Chai et al. developed and optimized a gene-editing strategy to correct *MYH7* c.1208G>A variant using multi-model approaches. First, CRISPR-Cas9 HDR was used to induce isogenic heterozygous and homozygous *MYH7* c.1208G>A variants into a healthy donor iPSC line, which was later used to screen for the highest-efficiency ABE enzyme. Next, iPSC-CMs were generated from HCM patient iPSC lines and subsequently targeted for correction using ABE_max_-VRQR, resulting in minimal bystander or off-target editing. Functional characterization of the iPSC-CM pathogenic *MYH7* variant alongside isogenic ABE_max_-VRQR-corrected lines demonstrated rescued hypercontractility and cellular energetics *in vitro.*^29^ These findings indicate the potential of base editing for preventing HCM phenotypes induced by well-known *MYH7* variants.

Moreover, the human iPSC-CM platform is also beneficial for exploring causation in rare variants not described in large population databases. For example, Yang et al. studied the uncommon *MYH7* p.E848G mutation that was found in an African American family but was not initially diagnosed as either HCM or DCM. Genome editing and viral transgenesis showed that the *MYH7* p.E848G variant had impaired contractile function, likely due to the impaired ability to bind cMyBP-C.^81^

The quality control mechanisms that maintain sarcomere integrity during mechanical stress of contraction are vital yet understudied. Pathogenic variants in alpha kinase 3 (*ALPK3*), an atypical kinase, provide a mechanistic link between phosphorylation and the removal of damaged sarcomere protein components in HCM. Patient-specific *ALPK3* variants (p.L639fs/34, p.Q1460X, and p.R1792X) were induced in human pluripotent stem cell-derived CMs to mimic a cohort of HCM patients.^82^ Cells with *ALPK3* patient-specific variants displayed sarcomere disarray with abnormal localization of M-band proteins, myomesin (MYOM1), and ubiquitin-binding protein sequestosome-1 (SQSTM1), suggesting that changes in the ALPK3-interactome mechanisms may underlie disease pathogenesis.^82^

### *Generation of iPSC-derived non-myocytes for disease modeling*

Although CMs occupy 70%–80% of the heart volume, they account for only a third of the total cell number. The surrounding non-myocyte populations are composed predominantly of CFs, ECs, SMCs, neurons, and immune cells.^83^ Understanding the complexities of cardiac cell-cell communication within the multicellular milieu is a critical consideration for the future of disease modeling and drug translation.

Resident CFs provide structural and mechanical maintenance in the heart through the extracellular matrix (ECM) network, which helps propagate conductivity and rhythmicity during contraction.^84^ During acute myocardial infarction, disease-activated fibroblasts play a role in the rapid deposition of ECM that prevents ventricular wall rupture, but leaves a fibrotic scar that is stiffer than a healthy myocardium. The accumulation of interstitial fibrosis coincident with myocardial stiffness, and associated with progressively worsening cardiac function, has been observed during chronic conditions of pathological remodeling.^85^ The clarification of the multifaceted roles of CFs in the maintenance of cardiac mechanosensation will likely continue to grow given our improving ability to derive iPSC-CFs from patients to model disease.^12^^,^^86^^,^^87^ For example, to understand genotype-phenotype differences in *LMNA-*associated dilated cardiomyopathies, iPSC-CFs and iPSC-CMs were differentiated from seven patients harboring missense *LMNA* mutations (p.M1L, p.R216C/R399H, p.R216C, p.R335Q, p.R377H, and p.R541C). Assessments of gene expression, signal transduction, and cellular morphology revealed phenotypically variable features beyond CM defects, thus underscoring a cell-intrinsic role for CFs in *LMNA*-DCM.^88^ Similarly, the incorporation of iPSC-CFs from patients with arrhythmogenic cardiomyopathy by plakophilin-2 (*PKP2*) c.2013delC heterozygous mutation into iPSC-derived cardiac microtissues displayed a myofibroblast-like phenotype, which weakened the conduction of iPSC-CMs during high-frequency pacing (≥2 Hz) and promoted arrhythmogenic behavior.^89^ Continued work using genetically defined co-culture thus expands our understanding of cellular interactomes surrounding resident CFs.

The vascular cells comprising the arteries, veins, capillaries, and lymphatic vessels are highly specialized, and the reconstruction of engineered vessels *in vitro* is challenging but has made significant progress in recent years. Today, human iPSCs can be differentiated into multiple vascular cell types, including iPSC-ECs, iPSC-SMCs, and iPSC-CPs that can recapitulate the functional hallmarks of primary cells. For example, a comparison of iPSC-ECs and pulmonary arterial ECs (PAECs) from patients with PA hypertension (PAH) showed similar responses to PAH therapies as measured by angiogenesis and apoptosis assays,^90^ thus providing a valuable starting material that facilitated the identification of potential drug candidates for PAH.^91^ Furthermore, iPSC-ECs and PAECs were used to predict drug-induced vascular toxicity by transcriptomic profiling after treatment with eight clinically relevant chemotherapeutic agents known to impair EC function.^92^

In a recent study, iPSC-ECs were used to uncover the pleiotropic activities of simvastatin that confer cardiovascular protection beyond cholesterol-lowering effects. Statins reduced chromatin accessibility of endothelial-to-mesenchymal transition-regulating genes in a yes-associated protein (YAP)-dependent manner, which rescued endothelial dysfunction under baseline and hyperglycemic conditions.^93^ The epigenetic regulation of the GGTase-RhoA-YAP1-SOX9 signaling axis thus provides a new therapeutic window for treating ischemic heart diseases that is mainly driven by atherosclerosis,^94^ the main contributor to global mortality.^95^

Coronary artery disease (CAD) is a multifactorial disorder influenced by the interplay between polygenic predispositions and environmental factors. ECs are critical structural components of blood vessels and crucial for maintaining vascular tone in the heart. Thus, coronary EC dysfunction is a well-recognized maladaptive process in the development of CAD from early initiation to end-stage atherothrombotic complication.^64^ Mutations in alcohol dehydrogenase 2 (*ALDH2*, also named *ALDH2∗2* rs671), which are prevalent in East Asian populations and manifest as an “alcohol flush,” are also associated with an increased risk of CAD.^64^^,^^96^ Using patient-derived *ALDH2∗2* rs671 iPSC-ECs, Guo et al. found increased oxidative and inflammatory markers alongside functionally impaired nitric oxide production and tube formation capacity. iPSC-EC function in *ALDH2∗2* rs671 was further exacerbated after ethanol exposure but was rescued using CRISPR-Cas9-mediated gene correction.^64^

Robust generation of iPSC-SMCs has expanded the scope of investigations of vascular diseases into rare genetic disorders of the aorta, such as MFS, which is caused by pathogenic variants in the critical ECM protein, fibrillin (*FBN1*). Patient-derived *FBN1* p.C1242Y iPSC-SMCs were found to recapitulate aspects of MFS^97^ (e.g., increased matrix metalloproteinase activity and apoptosis) as they underwent abnormal ECM deposition and response to mechanical stress, creating a platform for high-throughput phenotypic drug screening in the process.^66^

CPs play a key role in the maintenance, perfusion, and remodeling of the coronary vasculature, yet are an understudied cell population in the heart. Shen et al. demonstrated that iPSC-CPs can recapitulate hallmarks of primary cells, such as sunitinib-induced cytotoxicity that is attenuated by thalidomide.^14^ In addition, the integration of iPSC-CPs into multicellular organoids has increased our understanding of paracrine signaling in the microvessel network.^98^ Robust protocols for iPSC-CP generation will undoubtedly prove a useful tool for future exploration into genetic microvasculature malformations while serving as a platform for the development of proangiogenic cell therapies for myocardial infarction.^14^

### *Utilization of iPSC-derived EHTs and organoids for 3D cardiac modeling*

Toward the future goal of creating miniaturized multi-organ platforms, the ability of iPSC-derived cardiovascular cells to assemble into EHTs and organoids carries promise for higher complexity disease modeling (Figure 1). Recent studies have shown that iPSC-CMs assembled into EHTs could be used to model contractile deficits of RBM20 p.R636S DCM variant,^76^ validate the effects of combinatorial tyrosine kinase inhibitor treatment in improving contractility in pathogenic DCM variants,^65^ and assess the efficacy of diltiazem for HCM personalized care.^49^ Advancements in cardiac bioengineering methods continue to chip away at the limitations of 3D platforms, such as the tradeoff between throughput and maturity.^99^ Notably, the recent inclusion of non-myocyte cell populations enhanced the maturity and functionality of 3D cardiac microtissues^89^ and organoids^98^ by paracrine mechanisms. These strategies are now being applied to genetic studies, such as the use of cardiac organoids composed of healthy and cardiomyopathic (*MYH7* p.R719Q) iPSC-CMs that were combined with human cardiac microvascular ECs and human CFs.^100^ One consideration when using multi-cellular platforms for genetic studies is the laborious evaluation of each singular cell type, followed by the incremental addition of other cell types into the functional, 3D unit. Still, our ability to understand how a pathogenic variant influences its cellular interactome as well as inherent differences in cell-specific expression and cellular stoichiometry, will ultimately govern the efficacy of disease models moving forward.

### Limitations and future considerations for genetic disease in the heart

Most drug candidates fail to progress through clinical testing to approval, often due to the ineffectiveness of available disease models to predict potential toxicity or other issues.^101^^,^^102^ Human iPSC-based models have evolved rapidly in the past decade to bridge this clinical gap, but their full potential for precision medicine is only beginning to be unlocked.

A limitation to the utility of iPSC-CMs in clinical settings is their relative immaturity, as they structurally and functionally resemble fetal CMs.^103^^,^^104^ Molecular strategies to rapidly and robustly mature iPSC-CMs to generate mature sarcomere and ion channel protein expression are urgently needed for high-throughput drug- and patient-response predictions, and progress is being made on multiple fronts.^104^^,^^105^ Existing maturation protocols include physical stimulation (micropatterning, 3D aggregation, and mechanical manipulation^106^^,^^107^^,^^108^), biochemical stimulation (genetic and metabolic manipulation^103^^,^^109^), combinatorial approaches,^110^^,^^111^ and building multicellular platforms.^89^^,^^112^^,^^113^^,^^114^ Advances in novel methods and new iterations of existing strategies are expected to further clarify molecular mechanisms governing cardiac maturation and homeostasis.

Although current approaches are still rooted in iPSC-CM-autonomous processes, the research described here highlights the importance of working with other iPSC-derived cardiovascular cell types and integration into 3D-engineered tissue systems. We envision the incorporation of multicellular models that can allow cellular cross-talk will clarify masked or propagated aspects of genetic heart diseases previously unexplored.^99^^,^^115^

Precision medicine workflows consist of an arduous path from bedside to bench and back again, requiring substantial effort and resources. Still, these investments have proven fruitful for rare diseases, such as the rapid 1-year development and clinical deployment of a tailored oligonucleotide to treat a patient with Batten’s disease.^116^ This precedent fuels efforts toward tackling disease-causing mutations present in larger cohorts of patients. Further enhancing the predictive power of patient-derived iPSCs for personalized medicine is the integration of environmental factors, sex, and ethnicity within the genetic diversity of real-world patient populations.^58^^,^^117^ As new guidelines issued by the US Food and Drug Administration may limit animal use for drug testing in the future, increased reliance on human-based cell or organoid models for testing of cardiac toxicities is both likely and essential.^118^^,^^119^ Thus, new approaches to improve maturation strategies^120^^,^^121^ and assessments, as well as robust protocols to generate specialized cells and complex tissues, will be critical. Furthermore, early-phase iPSC-based drug screening platforms will benefit from the accessibility to growing patient biobanks,^122^^,^^123^ high-throughput functional pipelines,^124^ reporting tools,^125^ and small molecular libraries targeting key cardiovascular pathways.

## References

[1] Joseph P., Leong D., McKee M., Anand S.S., Schwalm J.-D., Teo K., Mente A., Yusuf S. (2017). Reducing the Global Burden of Cardiovascular Disease, Part 1. Circ. Res..

[2] Brooks I.R., Garrone C.M., Kerins C., Kiar C.S., Syntaka S., Xu J.Z., Spagnoli F.M., Watt F.M. (2022). Functional genomics and the future of iPSCs in disease modeling. Stem Cell Rep..

[3] Visscher P.M., Brown M.A., McCarthy M.I., Yang J. (2012). Five Years of GWAS Discovery. Am. J. Hum. Genet..

[4] Manolio T.A., Collins F.S., Cox N.J., Goldstein D.B., Hindorff L.A., Hunter D.J., McCarthy M.I., Ramos E.M., Cardon L.R., Chakravarti A. (2009). Finding the missing heritability of complex diseases. Nature.

[5] Litviňuková M., Talavera-López C., Maatz H., Reichart D., Worth C.L., Lindberg E.L., Kanda M., Polanski K., Heinig M., Lee M. (2020). Cells of the adult human heart. Nature.

[6] Hwang P.M., Sykes B.D. (2015). Targeting the sarcomere to correct muscle function. Nat. Rev. Drug Discov..

[7] Schwartz P.J., Ackerman M.J., Antzelevitch C., Bezzina C.R., Borggrefe M., Cuneo B.F., Wilde A.A.M. (2020). Inherited cardiac arrhythmias. Nat. Rev. Dis. Prim..

[8] Kamo T., Akazawa H., Komuro I. (2015). Cardiac Nonmyocytes in the Hub of Cardiac Hypertrophy. Circ. Res..

[9] Tian Y., Morrisey E.E. (2012). Importance of Myocyte-Nonmyocyte Interactions in Cardiac Development and Disease. Circ. Res..

[10] Lee J., Termglinchan V., Diecke S., Itzhaki I., Lam C.K., Garg P., Lau E., Greenhaw M., Seeger T., Wu H. (2019). Activation of PDGF pathway links LMNA mutation to dilated cardiomyopathy. Nature.

[11] Sayed N., Liu C., Ameen M., Himmati F., Zhang J.Z., Khanamiri S., Moonen J.-R., Wnorowski A., Cheng L., Rhee J.-W. (2020). Clinical trial in a dish using iPSCs shows lovastatin improves endothelial dysfunction and cellular cross-talk in LMNA cardiomyopathy. Sci. Transl. Med..

[12] Zhang H., Tian L., Shen M., Tu C., Wu H., Gu M., Paik D.T., Wu J.C. (2019). Generation of Quiescent Cardiac Fibroblasts From Human Induced Pluripotent Stem Cells for In Vitro Modeling of Cardiac Fibrosis. Circ. Res..

[13] Shen M., Quertermous T., Fischbein M.P., Wu J.C. (2021). Generation of Vascular Smooth Muscle Cells From Induced Pluripotent Stem Cells. Circ. Res..

[14] Shen M., Liu C., Zhao S.R., Manhas A., Sundaram L., Ameen M., Wu J.C. (2023). Stepwise Generation of Human Induced Pluripotent Stem Cell–Derived Cardiac Pericytes to Model Coronary Microvascular Dysfunction. Circulation.

[15] Wu J.C., Garg P., Yoshida Y., Yamanaka S., Gepstein L., Hulot J.-S., Knollmann B.C., Schwartz P.J. (2019). Towards Precision Medicine With Human iPSCs for Cardiac Channelopathies. Circ. Res..

[16] Brugada P., Brugada J. (1992). Right bundle branch block, persistent ST segment elevation and sudden cardiac death: A distinct clinical and electrocardiographic syndrome: A multicenter report. J. Am. Coll. Cardiol..

[17] Mizusawa Y., Wilde A.A.M. (2012). Brugada Syndrome. Circ. Arrhythm. Electrophysiol..

[18] Belbachir N., Portero V., Al Sayed Z.R., Gourraud J.-B., Dilasser F., Jesel L., Guo H., Wu H., Gaborit N., Guilluy C. (2019). RRAD mutation causes electrical and cytoskeletal defects in cardiomyocytes derived from a familial case of Brugada syndrome. Eur. Heart J..

[19] O’Neill M.J., Sala L., Denjoy I., Wada Y., Kozek K., Crotti L., Dagradi F., Kotta M.-C., Spazzolini C., Leenhardt A. (2023). Continuous Bayesian variant interpretation accounts for incomplete penetrance among Mendelian cardiac channelopathies. Genet. Med..

[20] Kozek K., Wada Y., Sala L., Denjoy I., Egly C., O’Neill M.J., Aiba T., Shimizu W., Makita N., Ishikawa T. (2021). Estimating the Posttest Probability of Long QT Syndrome Diagnosis for Rare KCNH2 Variants. Circ. Genom. Precis. Med..

[21] Kroncke B.M., Smith D.K., Zuo Y., Glazer A.M., Roden D.M., Blume J.D. (2020). A Bayesian method to estimate variant-induced disease penetrance. PLoS Genet..

[22] Kernik D.C., Yang P.-C., Kurokawa J., Wu J.C., Clancy C.E. (2020). A computational model of induced pluripotent stem-cell derived cardiomyocytes for high throughput risk stratification of KCNQ1 genetic variants. PLoS Comput. Biol..

[23] Serrano R., Feyen D.A.M., Bruyneel A.A.N., Hnatiuk A.P., Vu M.M., Amatya P.L., Perea-Gil I., Prado M., Seeger T., Wu J.C. (2023). A deep learning platform to assess drug proarrhythmia risk. Cell Stem Cell.

[24] Brumpton B.M., Graham S., Surakka I., Skogholt A.H., Løset M., Fritsche L.G., Wolford B., Zhou W., Nielsen J.B., Holmen O.L. (2022). The HUNT study: A population-based cohort for genetic research. Cell Genom..

[25] Nishiga M., Qi L.S., Wu J.C. (2021). Therapeutic genome editing in cardiovascular diseases. Adv. Drug Deliv. Rev..

[26] Liu N., Olson E.N. (2022). CRISPR Modeling and Correction of Cardiovascular Disease. Circ. Res..

[27] Hsu P.D., Lander E.S., Zhang F. (2014). Development and Applications of CRISPR-Cas9 for Genome Engineering. Cell.

[28] Nishiga M., Liu C., Qi L.S., Wu J.C. (2022). The use of new CRISPR tools in cardiovascular research and medicine. Nat. Rev. Cardiol..

[29] Chai A.C., Cui M., Chemello F., Li H., Chen K., Tan W., Atmanli A., McAnally J.R., Zhang Y., Xu L. (2023). Base editing correction of hypertrophic cardiomyopathy in human cardiomyocytes and humanized mice. Nat. Med..

[30] Reichart D., Newby G.A., Wakimoto H., Lun M., Gorham J.M., Curran J.J., Raguram A., DeLaughter D.M., Conner D.A., Marsiglia J.D.C. (2023). Efficient in vivo genome editing prevents hypertrophic cardiomyopathy in mice. Nat. Med..

[31] Anzalone A.V., Koblan L.W., Liu D.R. (2020). Genome editing with CRISPR–Cas nucleases, base editors, transposases and prime editors. Nat. Biotechnol..

[32] Nishiyama T., Zhang Y., Cui M., Li H., Sanchez-Ortiz E., McAnally J.R., Tan W., Kim J., Chen K., Xu L. (2022). Precise genomic editing of pathogenic mutations in RBM20 rescues dilated cardiomyopathy. Sci. Transl. Med..

[33] Chemello F., Chai A.C., Li H., Rodriguez-Caycedo C., Sanchez-Ortiz E., Atmanli A., Mireault A.A., Liu N., Bassel-Duby R., Olson E.N. (2021). Precise correction of Duchenne muscular dystrophy exon deletion mutations by base and prime editing. Sci. Adv..

[34] Wang P., Li H., Zhu M., Han R.Y., Guo S., Han R. (2023). Correction of DMD in human iPSC-derived cardiomyocytes by base-editing-induced exon skipping. Mol. Ther. Methods Clin. Dev..

[35] Vandemoortele G., De Sutter D., Moliere A., Pauwels J., Gevaert K., Eyckerman S. (2019). A Well-Controlled BioID Design for Endogenous Bait Proteins. J. Proteome Res..

[36] Schwinn M.K., Machleidt T., Zimmerman K., Eggers C.T., Dixon A.S., Hurst R., Hall M.P., Encell L.P., Binkowski B.F., Wood K.V. (2018). CRISPR-Mediated Tagging of Endogenous Proteins with a Luminescent Peptide. ACS Chem. Biol..

[37] Liu J., Jang J.Y., Pirooznia M., Liu S., Finkel T. (2021). The secretome mouse provides a genetic platform to delineate tissue-specific in vivo secretion. Proc. Natl. Acad. Sci. USA.

[38] Qin W., Cho K.F., Cavanagh P.E., Ting A.Y. (2021). Deciphering molecular interactions by proximity labeling. Nat. Methods.

[39] Branon T.C., Bosch J.A., Sanchez A.D., Udeshi N.D., Svinkina T., Carr S.A., Feldman J.L., Perrimon N., Ting A.Y. (2018). Efficient proximity labeling in living cells and organisms with TurboID. Nat. Biotechnol..

[40] Cho K.F., Branon T.C., Udeshi N.D., Myers S.A., Carr S.A., Ting A.Y. (2020). Proximity labeling in mammalian cells with TurboID and split-TurboID. Nat. Protoc..

[41] Roux K.J., Kim D.I., Raida M., Burke B. (2012). A promiscuous biotin ligase fusion protein identifies proximal and interacting proteins in mammalian cells. J. Cell Biol..

[42] Kushner J.S., Liu G., Eisert R.J., Bradshaw G.A., Pitt G.S., Hinson J.T., Kalocsay M., Marx S.O. (2022). Detecting Cardiovascular Protein-Protein Interactions by Proximity Proteomics. Circ. Res..

[43] Feng W., Liu C., Spinozzi S., Wang L., Evans S.M., Chen J. (2020). Identifying the Cardiac Dyad Proteome In Vivo by a BioID2 Knock-In Strategy. Circulation.

[44] Gonzalez-Teran B., Pittman M., Felix F., Thomas R., Richmond-Buccola D., Hüttenhain R., Choudhary K., Moroni E., Costa M.W., Huang Y. (2022). Transcription factor protein interactomes reveal genetic determinants in heart disease. Cell.

[45] Huynh K. (2022). Protein interactomes uncover new genetic causes of CHD. Nat. Rev. Cardiol..

[46] Peper J., Kownatzki-Danger D., Weninger G., Seibertz F., Pronto J.R.D., Sutanto H., Pacheu-Grau D., Hindmarsh R., Brandenburg S., Kohl T. (2021). Caveolin3 Stabilizes McT1-Mediated Lactate/Proton Transport in Cardiomyocytes. Circ. Res..

[47] Sjöblom B., Salmazo A., Djinović-Carugo K. (2008). α-Actinin structure and regulation. Cell. Mol. Life Sci..

[48] Ladha F.A., Thakar K., Pettinato A.M., Legere N., Ghahremani S., Cohn R., Romano R., Meredith E., Chen Y.-S., Hinson J.T. (2021). Actinin BioID reveals sarcomere crosstalk with oxidative metabolism through interactions with IGF2BP2. Cell Rep..

[49] Prondzynski M., Lemoine M.D., Zech A.T., Horváth A., Di Mauro V., Koivumäki J.T., Kresin N., Busch J., Krause T., Krämer E. (2019). Disease modeling of a mutation in α-actinin 2 guides clinical therapy in hypertrophic cardiomyopathy. EMBO Mol. Med..

[50] Lu L., Zhang Q., Timofeyev V., Zhang Z., Young J.N., Shin H.-S., Knowlton A.A., Chiamvimonvat N. (2007). Molecular Coupling of a Ca2+-Activated K+ Channel to L-Type Ca2+ Channels via Actinin2. Circ. Res..

[51] Eden M., Meder B., Völkers M., Poomvanicha M., Domes K., Branchereau M., Marck P., Will R., Bernt A., Rangrez A. (2016). Myoscape controls cardiac calcium cycling and contractility via regulation of L-type calcium channel surface expression. Nat. Commun..

[52] Zech A.T.L., Prondzynski M., Singh S.R., Pietsch N., Orthey E., Alizoti E., Busch J., Madsen A., Behrens C.S., Meyer-Jens M. (2022). ACTN2 Mutant Causes Proteopathy in Human iPSC-Derived Cardiomyocytes. Cells.

[53] Chiu C., Bagnall R.D., Ingles J., Yeates L., Kennerson M., Donald J.A., Jormakka M., Lind J.M., Semsarian C. (2010). Mutations in Alpha-Actinin-2 Cause Hypertrophic Cardiomyopathy: A Genome-Wide Analysis. J. Am. Coll. Cardiol..

[54] Theis J.L., Bos J.M., Bartleson V.B., Will M.L., Binder J., Vatta M., Towbin J.A., Gersh B.J., Ommen S.R., Ackerman M.J. (2006). Echocardiographic-determined septal morphology in Z-disc hypertrophic cardiomyopathy. Biochem. Biophys. Res. Commun..

[55] Girolami F., Iascone M., Tomberli B., Bardi S., Benelli M., Marseglia G., Pescucci C., Pezzoli L., Sana M.E., Basso C. (2014). Novel α-Actinin 2 Variant Associated With Familial Hypertrophic Cardiomyopathy and Juvenile Atrial Arrhythmias. Circ. Cardiovasc. Genet..

[56] Haywood N.J., Wolny M., Rogers B., Trinh C.H., Shuping Y., Edwards T.A., Peckham M. (2016). Hypertrophic cardiomyopathy mutations in the calponin-homology domain of ACTN2 affect actin binding and cardiomyocyte Z-disc incorporation. Biochem. J..

[57] Fordyce C.B., Roe M.T., Ahmad T., Libby P., Borer J.S., Hiatt W.R., Bristow M.R., Packer M., Wasserman S.M., Braunstein N. (2015). Cardiovascular Drug Development. J. Am. Coll. Cardiol..

[58] Hnatiuk A., Mercola M. (2019). Stars in the Night Sky: iPSC-Cardiomyocytes Return the Patient Context to Drug Screening. Cell Stem Cell.

[59] Burridge P.W., Li Y.F., Matsa E., Wu H., Ong S.-G., Sharma A., Holmström A., Chang A.C., Coronado M.J., Ebert A.D. (2016). Human induced pluripotent stem cell–derived cardiomyocytes recapitulate the predilection of breast cancer patients to doxorubicin-induced cardiotoxicity. Nat. Med..

[60] Lipshultz S.E., Cochran T.R., Franco V.I., Miller T.L. (2013). Treatment-related cardiotoxicity in survivors of childhood cancer. Nat. Rev. Clin. Oncol..

[61] Aminkeng F., Bhavsar A.P., Visscher H., Rassekh S.R., Li Y., Lee J.W., Brunham L.R., Caron H.N., van Dalen E.C., Kremer L.C. (2015). A coding variant in RARG confers susceptibility to anthracycline-induced cardiotoxicity in childhood cancer. Nat. Genet..

[62] Magdy T., Jiang Z., Jouni M., Fonoudi H., Lyra-Leite D., Jung G., Romero-Tejeda M., Kuo H.-H., Fetterman K.A., Gharib M. (2021). RARG variant predictive of doxorubicin-induced cardiotoxicity identifies a cardioprotective therapy. Cell Stem Cell.

[63] Briganti F., Sun H., Wei W., Wu J., Zhu C., Liss M., Karakikes I., Rego S., Cipriano A., Snyder M. (2020). iPSC Modeling of RBM20-Deficient DCM Identifies Upregulation of RBM20 as a Therapeutic Strategy. Cell Rep..

[64] Guo H., Yu X., Liu Y., Paik D.T., Justesen J.M., Chandy M., Jahng J.W.S., Zhang T., Wu W., Rwere F. (2023). SGLT2 inhibitor ameliorates endothelial dysfunction associated with the common ALDH2 alcohol flushing variant. Sci. Transl. Med..

[65] Perea-Gil I., Seeger T., Bruyneel A.A.N., Termglinchan V., Monte E., Lim E.W., Vadgama N., Furihata T., Gavidia A.A., Arthur Ataam J. (2022). Serine biosynthesis as a novel therapeutic target for dilated cardiomyopathy. Eur. Heart J..

[66] Davaapil H., McNamara M., Granata A., Macrae R.G.C., Hirano M., Fitzek M., Aragon-Martin J.A., Child A., Smith D.M., Sinha S. (2023). A phenotypic screen of Marfan syndrome iPSC-derived vascular smooth muscle cells uncovers GSK3β as a new target. Stem Cell Rep..

[67] Hnatiuk A.P., Briganti F., Staudt D.W., Mercola M. (2021). Human iPSC modeling of heart disease for drug development. Cell Chem. Biol..

[68] Hershberger R.E., Hedges D.J., Morales A. (2013). Dilated cardiomyopathy: the complexity of a diverse genetic architecture. Nat. Rev. Cardiol..

[69] McNally E.M., Golbus J.R., Puckelwartz M.J. (2013). Genetic mutations and mechanisms in dilated cardiomyopathy. J. Clin. Invest..

[70] Schultheiss H.-P., Fairweather D., Caforio A.L.P., Escher F., Hershberger R.E., Lipshultz S.E., Liu P.P., Matsumori A., Mazzanti A., McMurray J., Priori S.G. (2019). Dilated cardiomyopathy. Nat. Rev. Dis. Prim..

[71] Ahmed A., Syed J.N., Chi L., Wang Y., Perez-Romero C., Lee D., Kocaqi E., Caballero A., Yang J., Escalante-Covarrubias Q. (2023). KDM8 epigenetically controls cardiac metabolism to prevent initiation of dilated cardiomyopathy. Nat. Cardiovasc. Res..

[72] Jordan E., Peterson L., Ai T., Asatryan B., Bronicki L., Brown E., Celeghin R., Edwards M., Fan J., Ingles J. (2021). Evidence-Based Assessment of Genes in Dilated Cardiomyopathy. Circulation.

[73] McDermott-Roe C., Lv W., Maximova T., Wada S., Bukowy J., Marquez M., Lai S., Shehu A., Benjamin I., Geurts A., Musunuru K. (2019). Investigation of a dilated cardiomyopathy–associated variant in BAG3 using genome-edited iPSC-derived cardiomyocytes. JCI Insight.

[74] Takayama S., Xie Z., Reed J.C. (1999). An Evolutionarily Conserved Family of Hsp70/Hsc70 Molecular Chaperone Regulators. J. Biol. Chem..

[75] Norton N., Li D., Rieder M.J., Siegfried J.D., Rampersaud E., Züchner S., Mangos S., Gonzalez-Quintana J., Wang L., McGee S. (2011). Genome-wide Studies of Copy Number Variation and Exome Sequencing Identify Rare Variants in BAG3 as a Cause of Dilated Cardiomyopathy. Am. J. Hum. Genet..

[76] Fenix A.M., Miyaoka Y., Bertero A., Blue S.M., Spindler M.J., Tan K.K.B., Perez-Bermejo J.A., Chan A.H., Mayerl S.J., Nguyen T.D. (2021). Gain-of-function cardiomyopathic mutations in RBM20 rewire splicing regulation and re-distribute ribonucleoprotein granules within processing bodies. Nat. Commun..

[77] Kornienko J., Rodríguez-Martínez M., Fenzl K., Hinze F., Schraivogel D., Grosch M., Tunaj B., Lindenhofer D., Schraft L., Kueblbeck M. (2023). Mislocalization of pathogenic RBM20 variants in dilated cardiomyopathy is caused by loss-of-interaction with Transportin-3. Nat. Commun..

[78] Nemani L., Barik R., Patnaik A.N., Mishra R.C., Rao A.M., Kapur P. (2014). Coffin-Siris syndrome with the rarest constellation of congenital cardiac defects: A case report with review of literature. Ann. Pediatr. Cardiol..

[79] McNally E.M., Barefield D.Y., Puckelwartz M.J. (2015). The Genetic Landscape of Cardiomyopathy and Its Role in Heart Failure. Cell Metabol..

[80] Kensler R.W., Shaffer J.F., Harris S.P. (2011). Binding of the N-terminal fragment C0–C2 of cardiac MyBP-C to cardiac F-actin. J. Struct. Biol..

[81] Yang K.-C., Breitbart A., De Lange W.J., Hofsteen P., Futakuchi-Tsuchida A., Xu J., Schopf C., Razumova M.V., Jiao A., Boucek R. (2018). Novel Adult-Onset Systolic Cardiomyopathy Due to MYH7 E848G Mutation in Patient-Derived Induced Pluripotent Stem Cells. JACC. Basic Transl. Sci..

[82] McNamara J.W., Parker B.L., Voges H.K., Mehdiabadi N.R., Bolk F., Ahmad F., Chung J.D., Charitakis N., Molendijk J., Zech A.T.L. (2023). Alpha kinase 3 signaling at the M-band maintains sarcomere integrity and proteostasis in striated muscle. Nat. Cardiovasc. Res..

[83] Pinto A.R., Ilinykh A., Ivey M.J., Kuwabara J.T., D’Antoni M.L., Debuque R., Chandran A., Wang L., Arora K., Rosenthal N.A., Tallquist M.D. (2016). Revisiting Cardiac Cellular Composition. Circ. Res..

[84] Tallquist M.D., Molkentin J.D. (2017). Redefining the identity of cardiac fibroblasts. Nat. Rev. Cardiol..

[85] Pesce M., Duda G.N., Forte G., Girao H., Raya A., Roca-Cusachs P., Sluijter J.P.G., Tschöpe C., Van Linthout S. (2023). Cardiac fibroblasts and mechanosensation in heart development, health and disease. Nat. Rev. Cardiol..

[86] Zhang H., Shen M., Wu J.C., Nagy A., Turksen K. (2022). Induced Pluripotent Stem (iPS) Cells: Methods and Protocols.

[87] Zhang J., Tao R., Campbell K.F., Carvalho J.L., Ruiz E.C., Kim G.C., Schmuck E.G., Raval A.N., da Rocha A.M., Herron T.J. (2019). Functional cardiac fibroblasts derived from human pluripotent stem cells via second heart field progenitors. Nat. Commun..

[88] Yang J., Argenziano M.A., Burgos Angulo M., Bertalovitz A., Beidokhti M.N., McDonald T.V. (2021). Phenotypic Variability in iPSC-Induced Cardiomyocytes and Cardiac Fibroblasts Carrying Diverse LMNA Mutations. Front. Physiol..

[89] Giacomelli E., Meraviglia V., Campostrini G., Cochrane A., Cao X., van Helden R.W.J., Krotenberg Garcia A., Mircea M., Kostidis S., Davis R.P. (2020). Human-iPSC-Derived Cardiac Stromal Cells Enhance Maturation in 3D Cardiac Microtissues and Reveal Non-cardiomyocyte Contributions to Heart Disease. Cell Stem Cell.

[90] Gu M., Shao N.-Y., Sa S., Li D., Termglinchan V., Ameen M., Karakikes I., Sosa G., Grubert F., Lee J. (2017). Patient-Specific iPSC-Derived Endothelial Cells Uncover Pathways that Protect against Pulmonary Hypertension in BMPR2 Mutation Carriers. Cell Stem Cell.

[91] Gu M., Donato M., Guo M., Wary N., Miao Y., Mao S., Saito T., Otsuki S., Wang L., Harper R.L. (2021). iPSC–endothelial cell phenotypic drug screening and in silico analyses identify tyrphostin-AG1296 for pulmonary arterial hypertension. Sci. Transl. Med..

[92] Tu C., Liu Y., Williams D.R., Wu J.C. (2022). A transcriptomic atlas of drug-induced endothelial dysfunction in human endothelial cells. J. Mol. Cell. Cardiol..

[93] Liu C., Shen M., Tan W.L.W., Chen I.Y., Liu Y., Yu X., Yang H., Zhang A., Liu Y., Zhao M.-T. (2023). Statins improve endothelial function via suppression of epigenetic-driven EndMT. Nat. Cardiovasc. Res..

[94] Bae S., Jung C., Yoon Y.-s. (2023). Rescue of EndMT-associated endothelial dysfunction by modulating the YAP pathway. Nat. Cardiovasc. Res..

[95] Libby P. (2021). The changing landscape of atherosclerosis. Nature.

[96] Li Y.-y., Wang H., Wu J.-j., Kim H.J., Yang X.-x., Geng H.-y., Gong G. (2018). ALDH2 gene G487A polymorphism and coronary artery disease: a meta-analysis including 5644 participants. J. Cell Mol. Med..

[97] Granata A., Serrano F., Bernard W.G., McNamara M., Low L., Sastry P., Sinha S. (2017). An iPSC-derived vascular model of Marfan syndrome identifies key mediators of smooth muscle cell death. Nat. Genet..

[98] Voges H.K., Foster S.R., Reynolds L., Parker B.L., Devilée L., Quaife-Ryan G.A., Fortuna P.R.J., Mathieson E., Fitzsimmons R., Lor M. (2023). Vascular cells improve functionality of human cardiac organoids. Cell Rep..

[99] Cho S., Discher D.E., Leong K.W., Vunjak-Novakovic G., Wu J.C. (2022). Challenges and opportunities for the next generation of cardiovascular tissue engineering. Nat. Methods.

[100] Filippo Buono M., von Boehmer L., Strang J., Hoerstrup S.P., Emmert M.Y., Nugraha B. (2020). Human Cardiac Organoids for Modeling Genetic Cardiomyopathy. Cells.

[101] Horvath P., Aulner N., Bickle M., Davies A.M., Nery E.D., Ebner D., Montoya M.C., Östling P., Pietiäinen V., Price L.S. (2016). Screening out irrelevant cell-based models of disease. Nat. Rev. Drug Discov..

[102] Matsa E., Burridge P.W., Wu J.C. (2014). Human Stem Cells for Modeling Heart Disease and for Drug Discovery. Sci. Transl. Med..

[103] Tu C., Chao B.S., Wu J.C. (2018). Strategies for Improving the Maturity of Human Induced Pluripotent Stem Cell-Derived Cardiomyocytes. Circ. Res..

[104] Caudal A., Ren L., Tu C., Wu J.C. (2022). Human induced pluripotent stem cells for studying mitochondrial diseases in the heart. FEBS Lett..

[105] Thomas D., Cunningham N.J., Shenoy S., Wu J.C. (2022). Human-induced pluripotent stem cells in cardiovascular research: current approaches in cardiac differentiation, maturation strategies, and scalable production. Cardiovasc. Res..

[106] Ebert A., Joshi A.U., Andorf S., Dai Y., Sampathkumar S., Chen H., Li Y., Garg P., Toischer K., Hasenfuss G. (2019). Proteasome-Dependent Regulation of Distinct Metabolic States During Long-Term Culture of Human iPSC-Derived Cardiomyocytes. Circ. Res..

[107] Shadrin I.Y., Allen B.W., Qian Y., Jackman C.P., Carlson A.L., Juhas M.E., Bursac N. (2017). Cardiopatch platform enables maturation and scale-up of human pluripotent stem cell-derived engineered heart tissues. Nat. Commun..

[108] Strimaityte D., Tu C., Yanez A., Itzhaki I., Wu H., Wu J.C., Yang H. (2022). Contractility and Calcium Transient Maturation in the Human iPSC-Derived Cardiac Microfibers. ACS Appl. Mater. Interfaces.

[109] Feyen D.A.M., McKeithan W.L., Bruyneel A.A.N., Spiering S., Hörmann L., Ulmer B., Zhang H., Briganti F., Schweizer M., Hegyi B. (2020). Metabolic Maturation Media Improve Physiological Function of Human iPSC-Derived Cardiomyocytes. Cell Rep..

[110] Huang C.Y., Peres Moreno Maia-Joca R., Ong C.S., Wilson I., DiSilvestre D., Tomaselli G.F., Reich D.H. (2020). Enhancement of human iPSC-derived cardiomyocyte maturation by chemical conditioning in a 3D environment. J. Mol. Cell. Cardiol..

[111] Abilez O.J., Tzatzalos E., Yang H., Zhao M.-T., Jung G., Zöllner A.M., Tiburcy M., Riegler J., Matsa E., Shukla P. (2018). Passive Stretch Induces Structural and Functional Maturation of Engineered Heart Muscle as Predicted by Computational Modeling. Stem Cell..

[112] Giacomelli E., Bellin M., Sala L., van Meer B.J., Tertoolen L.G.J., Orlova V.V., Mummery C.L. (2017). Three-dimensional cardiac microtissues composed of cardiomyocytes and endothelial cells co-differentiated from human pluripotent stem cells. Development.

[113] Tiburcy M., Hudson J.E., Balfanz P., Schlick S., Meyer T., Chang Liao M.L., Levent E., Raad F., Zeidler S., Wingender E. (2017). Defined Engineered Human Myocardium With Advanced Maturation for Applications in Heart Failure Modeling and Repair. Circulation.

[114] Mills R.J., Titmarsh D.M., Koenig X., Parker B.L., Ryall J.G., Quaife-Ryan G.A., Voges H.K., Hodson M.P., Ferguson C., Drowley L. (2017). Functional screening in human cardiac organoids reveals a metabolic mechanism for cardiomyocyte cell cycle arrest. Proc. Natl. Acad. Sci. USA.

[115] Kim H., Kamm R.D., Vunjak-Novakovic G., Wu J.C. (2022). Progress in multicellular human cardiac organoids for clinical applications. Cell Stem Cell.

[116] Kim J., Hu C., Moufawad El Achkar C., Black L.E., Douville J., Larson A., Pendergast M.K., Goldkind S.F., Lee E.A., Kuniholm A. (2019). Patient-Customized Oligonucleotide Therapy for a Rare Genetic Disease. N. Engl. J. Med..

[117] Warren C.R., Jaquish C.E., Cowan C.A. (2017). The NextGen Genetic Association Studies Consortium: A Foray into In Vitro Population Genetics. Cell Stem Cell.

[118] Wadman M. (2023). FDA no longer has to require animal testing for new drugs. Science.

[119] Moutinho S. (2023). Researchers and regulators plan for a future without lab animals. Nat. Med..

[120] Miklas J.W., Clark E., Levy S., Detraux D., Leonard A., Beussman K., Showalter M.R., Smith A.T., Hofsteen P., Yang X. (2019). TFPa/HADHA is required for fatty acid beta-oxidation and cardiolipin re-modeling in human cardiomyocytes. Nat. Commun..

[121] Yang X., Rodriguez M.L., Leonard A., Sun L., Fischer K.A., Wang Y., Ritterhoff J., Zhao L., Kolwicz S.C., Pabon L. (2019). Fatty Acids Enhance the Maturation of Cardiomyocytes Derived from Human Pluripotent Stem Cells. Stem Cell Rep..

[122] Caudal A., Mondejar-Parreño G., Vera C.D., Williams D.R., Shenoy S.P., Liang D., Wu J.C. (2022). Generation of human induced pluripotent stem cell lines carrying heterozygous PLN mutation from dilated cardiomyopathy patients. Stem Cell Res..

[123] Kong X., Belbachir N., Zeng W., Yan C.D., Navada S., Perez M.V., Wu J.C. (2023). Generation of two induced pluripotent stem cell lines from catecholaminergic polymorphic ventricular tachycardia patients carrying RYR2 mutations. Stem Cell Res..

[124] Mills R.J., Parker B.L., Quaife-Ryan G.A., Voges H.K., Needham E.J., Bornot A., Ding M., Andersson H., Polla M., Elliott D.A. (2019). Drug Screening in Human PSC-Cardiac Organoids Identifies Pro-proliferative Compounds Acting via the Mevalonate Pathway. Cell Stem Cell.

[125] Zhang J.Z., Termglinchan V., Shao N.-Y., Itzhaki I., Liu C., Ma N., Tian L., Wang V.Y., Chang A.C.Y., Guo H. (2019). A Human iPSC Double-Reporter System Enables Purification of Cardiac Lineage Subpopulations with Distinct Function and Drug Response Profiles. Cell Stem Cell.

